# The New Era of Salivaomics in Dentistry: Frontiers and Facts in the Early Diagnosis and Prevention of Oral Diseases and Cancer

**DOI:** 10.3390/metabo12070638

**Published:** 2022-07-12

**Authors:** Flavia Papale, Simona Santonocito, Alessandro Polizzi, Antonino Lo Giudice, Saverio Capodiferro, Gianfranco Favia, Gaetano Isola

**Affiliations:** 1 Department of General Surgery and Surgical-Medical Specialties, School of Dentistry, University of Catania, Via S. Sofia 78, 95124 Catania, Italy; flavia.papale@yahoo.it (F.P.); alexpoli354@gmail.com (A.P.); antonino.logiudice@unict.it (A.L.G.); gaetano.isola@unict.it (G.I.); 2 Department of Interdisciplinary Medicine, University of Bari Aldo Moro, Policlinico-Piazza G. Cesare, 11, 70124 Bari, Italy; capodiferro.saverio@gmail.com (S.C.); gianfranco.favia@uniba.it (G.F.)

**Keywords:** salivaomics, saliva, periodontitis, salivary biomarkers, proteomics, oral disease, microbiome

## Abstract

Nowadays, with the development of new and highly sensitive, blood is not the only medium of choice for the diagnosis of several diseases and pathological conditions. Saliva is now considered a safe and non-invasive sample to study oral and systemic diseases, showing great diagnostic potential. According to several recent studies, saliva has emerged as an emerging biofluid for the early diagnosis of several diseases, indicated as a mirror of oral and systemic health and a valuable source of clinically relevant information. Indeed, several studies have observed that saliva is useful for detecting and diagnosing malignant tumours, human immunodeficiency virus, heart disease, and autoimmune diseases. The growing realisation that saliva is an inexhaustible source of information has led to the coining of the term ‘Salivaomics’, which includes five “omics” in connection with the main constituents of saliva: genome and epigenome, transcriptomics, metabolomics, lipidomics, proteomics and microbiota. All those may be changed by disease state, so they offer significant advantages in the early diagnosis and prognosis of oral diseases. The aim of the present review isto update and highlight the new frontiers of salivaomics in diagnosing and managing oral disorders, such as periodontitis, premalignant disorders, and oral squamous cell carcinoma (OSCC).

## 1. Introduction

There are different steps for disease diagnosis: anamnesis, physical examination and chemical analysis that allows to quantify specific cells and molecules in a biological sample [1]. Given that many disorders are undetectable before symptoms occur, there is a need to find new potential biomarkers. The biomarkers are molecules that reflect the patient’s state of health, and they are genetic material and their products [2].

In the twenty-first century, blood remains the first laboratory diagnostic sample, but not the only one, in fact, other biological fluids are gaining a great diagnostic relevance, such as saliva. Saliva has many advantages in collection compared to blood since it is non-invasively, simple and rapid to sample, making a perfect diagnostic biofluid also in children and elderly persons who often are not fully cooperative [3]. On the other hand, the main disadvantages of saliva samples are: discrepancy between serum and saliva marker’s levels, the salivary composition can be changed by the method of collection and the salivary flow [4]. These features are important in some systemic diseases like Sjogren’s syndrome or Cystic fibrosis [3], and the presence of enzymes that can alter the concentration of some diagnostic markers [4].

Currently, we are in the “emerging era of high-integrated precision diagnostics” [5]. Salivary diagnostic is an area constantly evolving that represents a fundamental diagnostic tool and helps clinicians make therapeutic choices [1].

**Figure 1:**
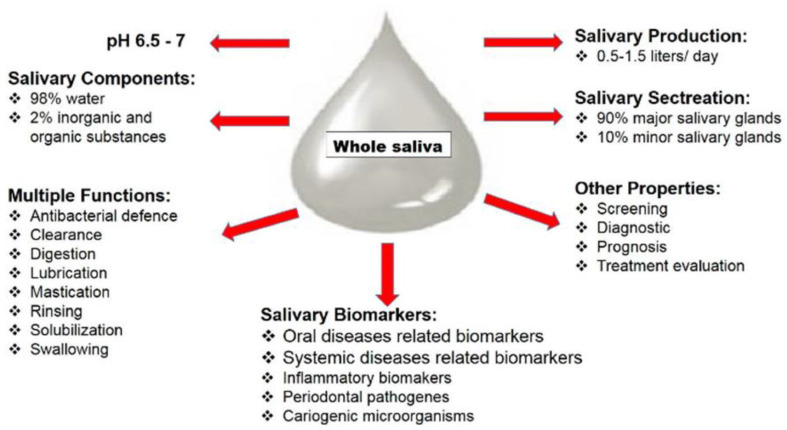
Saliva’s properties and composition. The human body produces, under normal conditions, between 0.5 and 1.5 L of saliva. It consists of 98% water and 2% various electrolytes, mucus, bacterial compounds and other enzymes. Saliva, like every human biofluid, performs a number of important functions related to its chemical and physical properties: rinsing, solubilization of food substances, elimination of food and bacteria, lubrication of soft tissue, bolus formation, dilution of de-bris, swallowing, speech and facilitation of chewing, coating of mucous membranes, digestion and antibacterial defense. Finally, saliva contains numerous biomarkers involved in the development of various systemic and oral diseases.

Whole saliva is a biofluid resulting from major and minor salivary glandular secretions, gingival crevicular fluid (GCF), expectorated bronchial, nasal secretions, bacteria, viruses, fungi and their products, desquamated epithelial cells and other cellular components [3]. Therefore, the saliva is a source of biomarkers also shared with the blood: hormones, antibodies, growth factors, enzymes, microbes and their products [2,6] (Figure 1).

**Figure 2:**
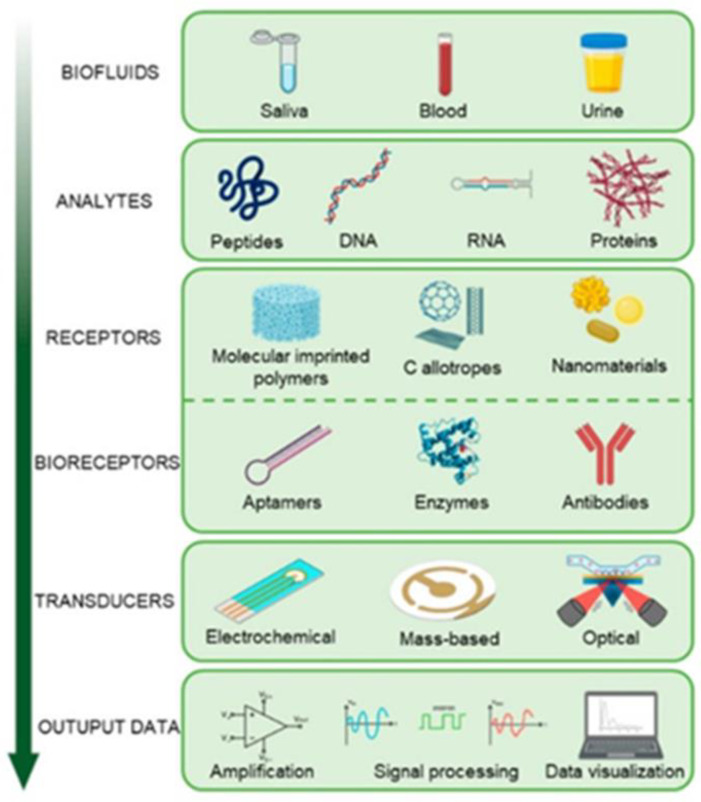
Biosensors’ operating and constituents. Biosensor systems are composed of three basic components. These are ‘biomolecule/bioagent’ with a mechanism of selective detection, ‘converter’ and ‘electronic’ parts capable of transforming the physical-chemical signals resulting from the interaction of this bioagent with the substance under investigation into an electronic signal.

The salivary diagnostic is becoming a clinical challenge may be attributed to developing innovative technologies capable of detecting small quantities, such as next-generation sequencing, proteomics, mass spectrometry, genome-wide association studies, and other screening techniques. Among the methods that have attracted considerable interest are biosensors for salivary diagnostics (Figure 2). They are still an area of research to be explored in order to understand their real utility compared to the techniques traditionally used in the study of biomarkers, including an established gold standard such as the ELISA test [7].

The study of salivary biomarkers allows the detection of salivary gland disorders like infection and obstruction and local and systemic diseases [8].

This review aims to analyze the existing role of salivomics in the identification of biomarkers useful in the early diagnosis and prevention of periodontal disease, premalignant disorders, and oral cell squamous carcinoma (OSCC).

## 2. Salivaomics in Dentistry

The term “salivaomics” was established in 2008 to reflect the rapid development of knowledge about saliva’s various “omics” constituents. Proteome, transcriptome, miRNA, metabolome and microbiome terms are closely related to saliva, and they offer the possibility to use saliva for clinical application during health and disease [9]. The association of emerging biotechnologies and salivary diagnostics makes it possible to study numerous molecules that reflect local and systemic diseases such as cancer, autoimmune diseases, bacterial diseases, and viral diseases. Salivaomics will allow physicians better management of disease therapy [10]. A huge amount of salivaomics data has been generated with the use of high-throughput technologies, so there was a need to have salivary data to collect biomarkers from different studies [9].

Denny et al. in 2008, began the human salivary proteome project to list all identified proteins in saliva with mass spectrometric approaches. Data from this study form part of the only salivary omics database available on the web [11]. In facts, the researchers of the W Lab at UCLA school of dentistry are pioneers of research on the use of biofluids as a diagnostic tool for early diagnosis and monitoring of oral and systemic disease. 

### 2.1. Proteomics

Proteomics is the analysis through high-throughput technology of all proteins, their expression, alterations and interactions [12]. Human salivary proteome analysis allows for investigating the possible presence of oral disorders and their pathogenesis [10]. The fields of application of proteomics are numerous, in fact, it is a branch shared by dentistry and other areas of medicine: it is used for the diagnosis of oral disorders, oral candidiasis, OSCC, glossodynia, head and neck squamous cell cancer, Sjogren’s syndrome, HIV, fibromyalgia, breast cancer, lung cancer, melanoma and pancreatic cancer [13].

The most currently used and validated technology in the study of proteomics is mass spectrometry (MS), supported by bioinformatics tools for data acquisition and management [14,15]. Through the technological advances, mass spectrometry platforms have switched from DDA (Data-Dependent Acquisition) mode to DIA (Data-Independent Acquisition) mode. In particular, the DDA mode carefully detects only the most abundant peptides and misses the others, while the DIA mode also allows the detection of traces of often significant peptides and biomarkers [16]. In addition to the two previously described modes, the targeted acquisition mass spectrometry strategies SRM (selected reaction moni-toring), MRM (multiple reaction monitoring) and PRM (parallel reaction monitoring), are techniques which can be effectively used for the precise and reproducible quantification of hundreds of low-abundance proteins [16]. The study of proteomics makes use of additional classical biochemical techniques, including gel electrophoresis, liquid chromatography and microarrays, which are used for sample stabilisation, fractionation and enrichment for protein groups or modifications prior to analysis by mass spectrometry [17,18]. Although the traditional methods described boast moderate sensitivity and specificity, their use has several disadvantages that make them impractical: the need for expensive equipment and highly skilled personnel; the need to collect, transfer and pre-treat samples with a high possibility of qualitatively altering or degrading some sample components; and poor reproducibility [19]. Sample pretreatment is performed to overcome the problem of rapid protein degradation by adding a protease inhibitor cocktail (PIC) to saliva samples. In [20] an attempt to overcome these disadvantages, various techniques using immunological tests have been introduced, which make it possible to overcome a major limitation of MS and classical biochemical techniques: the distortion of results in favour of highly abundant proteins over those with medium to low abundance [21,22]. New techniques have become increasingly popular in this context, including proximity extension technology (PEA) and aptamer-based techniques. PEA is a 96 plex immunoassay used for high-throughput detection of protein biomarkers in body fluids. In it, matched pairs of antibodies labelled with oligonucleotides bind to their target antigens pair-wise. After binding of the antibody, the corresponding oligonucleotides are brought together and, with the use of a DNA polymerase, a PCR target sequence is created, amplified, detected and quantified [23,24]. Aptamers are synthetic molecules consisting of short DNA or RNA strands that can bind a specific biological target (protein, virus, cell), altering its structural and functional characteristics. They can also be used as substitutes for antibodies in immunohistochemistry analyses or in ELISA assays to analyse blood, saliva and tissue samples by Western blot, microarray, fluorescence microscopy, confocal and X-ray. Another field of application for aptamers is the so-called ‘mix and measure’ method, which is capable of detecting very small amounts of analyte in a simple manner and without the need for washing and separation. By combining the specific binding properties of aptamers with the sensitivity of quantitative ‘real time PCR’, it is possible to detect very low concentrations of the analyte, which corresponds to a sensitivity a thousandfold higher than that of normal ELISA assays. Since aptamers can be easily bound to a very wide variety of markers (small radioactives, enzymes, etc.), many analytical applications can be designed, including biosensors on aptamers binding quenched fluorophores called aptamer beacons [25].

### 2.2. Transcriptomics

Transcriptomics reflects the genome’s functional elements because it is the set of all RNA transcripts [26]. It is an important source of potentially relevant diagnostic information, as it includes highly specific discriminatory indicators for several diseases like oral cancer, Sjogren’s syndrome, pancreatic cancer, lung cancer, ovarian cancer and breast cancer [13].

Several studies have indicated that human saliva comprises over 1000 miRNAs and more than 3000 mRNA species, of which 180 are common among several healthy participants, representing the normal core of the salivary transcriptome. Over the past few years, miRNAs have attracted considerable interest from the scientific community, which may even constitute a diagnostic alphabet in saliva in its own right [8]. Micro-RNAs (miRNAs) are short single-stranded, non-coding RNA sequences that result in post-transcriptional gene silencing [27]. They consist of 18–22 nucleotides, which bind to complementary sequences in the coding or 3′ untranslated region of target messenger RNAs, blocking translation or inducing degradation of target mRNAs [28]. Scientific evidence suggests that inhibition of protein synthesis by miRNAs is implicated in several physiological and pathological mechanisms. Indeed, it has been observed that aberrant expression of miRNAs leads to dysregulation of cellular responses involved in innate and adaptive immune responses, contributing to the development of chronic inflammatory diseases and cancer [29]. Therefore, miRNAs have a high diagnostic, prognostic and therapeutic potential and research has focused on finding possible miRNAs as biomarkers in saliva and gingival crevicular fluid of oral diseases and beyond [27]. Several analysis techniques are currently exploited to study miRNAs and transcriptomics more generally: microarrays, miRNAs sequencing (miRNA-seq), and reverse transcriptase-polymerase chain reaction (RT-PCR) [30]. Microarray technology has become an important tool for global gene expression and biomedical research in all aspects of human diseases [31]. However, the microarray technique has some limitations in miRNA expression analysis due to certain properties of miRNAs; the short length of miRNAs offers little sequence for optimisation of hybridisation efficiency. The large difference in GC content leads to very different hybridisation properties; the low abundance of some miRNAs; closely related miRNA family members differ even within a single nucleotide [32]. To overcome these problems, nucleotide analogues with more efficient hybridisation characteristics have been designed, such as locked nucleic acid oligonucleotides improve the melting temperature (Tm) of capture probes, enabling sensitive profiling of small RNAs [33]. The development of high-throughput next-generation sequencing (NGS) has revolutionised transcriptomics by enabling RNA analysis through complementary DNA (cDNA) sequencing. This method has distinct advantages over previous approaches, such as the microarray technique, providing a more detailed and quantitative view [34]. Finally, RT-PCR, a variant of the polymerase chain reaction (PCR) technique, allows the synthesis of a double-stranded DNA molecule from an RNA template. Using RT-PCR, it is possible to convert an entire transcriptome (set of all the transcripts of a cell) of a specific tissue of an individual at a specific stage of its development into DNA and study its gene expression [35].

### 2.3. Metabolome

The metabolome is the complete set of small molecular metabolites produced by metabolism. These molecules are in biological samples like saliva, and a change in their concentration can express diseases of dental interest such as oral cancer and periodontal disease [36]. Therefore, the parallel evaluation of a group of endogenous and exogenous metabolites, including lipids, amino acids, peptides, nucleic acids, organic acids, vitamins, thiols and carbohydrates, is a valuable tool for discovering biomarkers monitoring physiological status and making appropriate treatment decisions [8]. For example, taurine and piperidine are considered specific diagnostic markers of oral cancer [37].

### 2.4. Microbiome

The microbiome probably is one of the most interesting fields of salivaomic because there are about 19,000 microorganisms in saliva which affect the oral environment, in fact nearly 70% of the genome in the saliva is human; the remaining 30% belongs to oral microbiota. Consequently, oral dysbiosis can lead to oral diseases like periodontitis, caries or cancer [38]. In consideration of its ever-increasing importance and complexity, the relationship between saliva, the oral microbiome and oral diseases has been opened up in the following section.

## 3. Salivaomics and Oral Microbiome

Joshua Lederbeg coined the term microbiome “to signify the ecological community of commensal, symbiotic and pathogenic microorganisms that literally share our body space and have been all but ignored as determinants of health and disease” [39].

This definition highlights the need to study the oral microbiome because of its importance in health, disease and prevention. For this reason, the expanded Human Oral Microbiome Database (eHOMD) has been created, and it includes a total of 775 different microbial species, to provide the scientific community with comprehensive curated information on the bacterial species present in the human aerodigestive tract [40]. 

**Figure 3:**
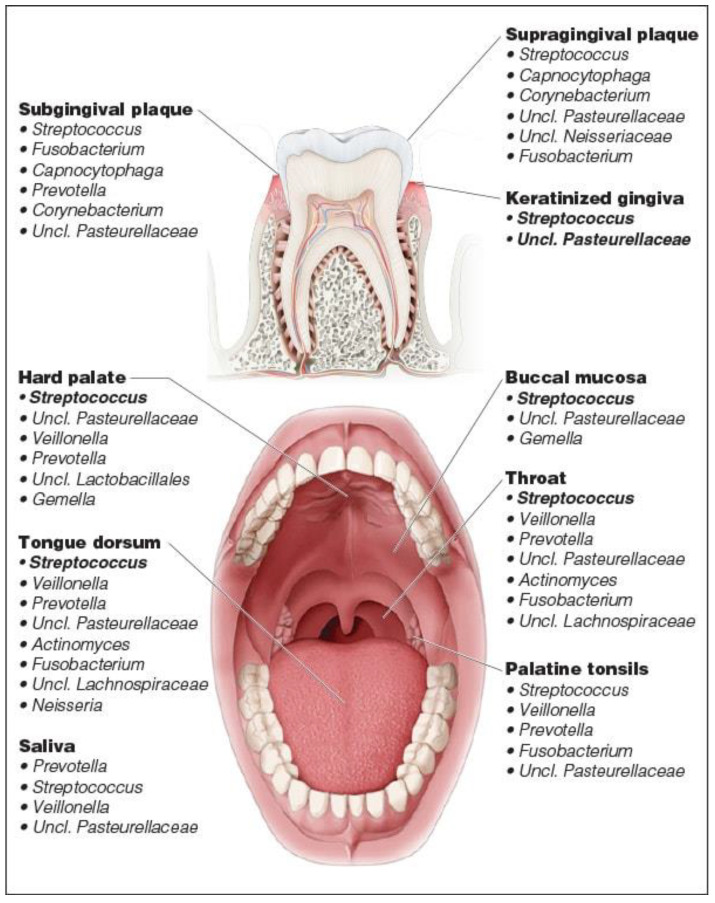
Microbial population in the oral cavity.

**Figure 4:**
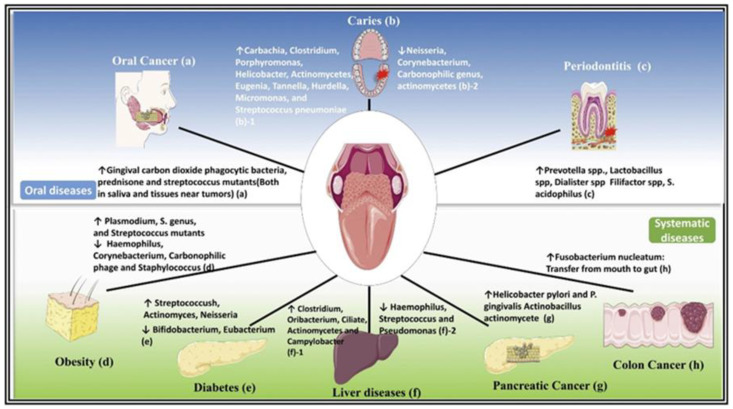
The oral microbiota is related to oral and systemic diseases. The oral microbiota is altered during oral and whole-body diseases. Therefore, the oral microbiota will be a new target for the treatment of oral diseases and the improvement of the body’s physical state. Reproduced with permission from Sampaio-Maia et al. [48].

The oral cavity represents an ideal environment for the growth of the microorganisms, in fact the 37 °C temperature of the oral cavity and pH 6.5 to 7.5 of saliva offer a permanent and ideal habitat for bacterial species [41]. However, saliva is sterile when it is secreted into the oral environment, but it is immediately colonized by bacteria shed from oral surfaces [42]. The oral microbiome consists proportionally of several bacterial phyla, of which the predominant ones are *Firmicutes*, *Proteobacteria*, *Bacteroidetes*, *Actinobacteria* and Fusobacteria [8]. Despite the inter-individual diversity, *Streptococcus* is commonly observed as the dominant genus in the healthy oral microbiome and *Prevotella*, *Veillonella*, *Neisseria* and *Haemophilus*. Interestingly, the composition of the oral microbiota is variable in relation to the intra-oral habitat analysed, reflecting the different properties of the surface and microenvironment (tooth surface, lateral and dorsal surface of the tongue [43] (Figure 3). In recent years, taking advantage of the development of new electromigration techniques (two-dimensional gel electrophoresis, capillary zone electrophoresis) and MS methods, such as matrix-assisted laser desorption time-of-flight (MALDI-TOF MS). These methods made it possible to overcome the limitations of classical molecular biology techniques: 16S ribosomal RNA (rRNA) gene sequencing, polymerase chain reaction (PCR) and other PCR-based methods. MALDI-TOF MS is a rapid and accurate method based on intact ionizing cells of microorganisms with short laser pulses and then accelerating the particles in a vacuum using an electric field. Each microorganism has a specific spectral profile [44]. Therefore, a better understanding of the composition of the oral microbiota, both under eubiose and dysbiosis conditions, has enabled a better understanding of the development and progression of certain systemic and oral diseases, including periodontitis caries and OSCC (Figure 4) [45,46,47]. 

For these reasons, saliva and its constituents are fundamental to ensuring a stable oral microbiome and, therefore a healthy mouth, though the complex interaction between host, saliva and oral microbiota; in fact saliva, with its composition, is an important nutritional source for many microorganisms. Therefore, a reduced salivary flow leads to dysbiosis and oral disease [48,49].

Since the microbial community can survive in sessile biofilm, but saliva contains mostly the planktonic members of the oral microbiome [42], the question is if saliva is a good microbial sample to study periodontitis and oral disease.

Wirth et al. have compared saliva samples, microbial samples from crevicular fluid and subgingival (plaque) samples. The comparison of the microbiomes of the same subjects with the different samples, highlights that saliva can give only “a fuzzy picture of the oral microbiome”. However, the most abundant microbes identified in the subgingival biofilm were also present in saliva samples, but in different concentrations, in fact Tannarella can represent an indicator genus in the periodontal pocket samples; on the other hand, Prevotella seems to be an indicator genus of the oral inflammation in saliva [50].

It is known that there are a lot of bacteria involved in periodontitis: *Porphyromonas gingivalis* (*P. gingivalis*), *Tannerella forsythia* (*T. forsythia*), *Treponema denticola* (*T. denticola*), *Filifactor alocis* (*F. alocis*), and *Peptoanaerobacter stomatis*, but *P. gingivalis*, *T. forsythia*, and *T. denticola* represent the main pathogens in chronic periodontitis [51]. In fact, periodontal disease results from microbiome’s dysbiosis that compromises the immune system, leading to a destructive inflammatory process [52]. Some bacterial species, particularly red-complex bacteria *T. forsythia*, *T. denticola*, and *P. gingivalis*, have been accepted as etiological agents of periodontal disease [53]. It has been demonstrated that exist a microbial interaction in the pathogenesis of periodontal disease, e.g. the metabolism products of S. gordonii can increase the virulence of *Aggregatibacter actinomycetemcomitans* (*A. actinomycetemcomitans*); *T. denticola* finally producing metabolites that improve the growth of *P. gingivalis* [54]. 

In addition to “red complex” bacteria, *F. alocis* is considered a key pathogenic bacteria in altering oral microbiome in aggressive periodontitis, so it also represents a periodontal disease biomarker [55]. Furthermore, lots of studies have proved that *Porphyromonas endodontalis*, *Eubacterium saphenum*, *Eubacterium branchy* and *F. alocis* are present in the most severe periodontal pockets, therefore, they are related to the severity of the disease [56].

Moreover, bacteria do not only represent biomarkers for periodontitis but also for oral cancer, in fact both *P. gingivalis* and *Fusobacterium Nucleatum* have carcinogenic potential demonstrated in vitro as well as in vivo model [51]. It has been observed an increased salivary concentration of *Capnocytophaga gingivalis*, *Prevotella melaninogenica* and *Streptococcus mitis* in oral cancer affected patients compared to healthy controls. These bacterial species showed an 80% sensitivity and a 83% specificity as diagnostic biomarkers for the differentiation between controls and cases [57]. Growing interest is emerging in the scientific community for the study of how alterations in the oral microbiome can alter the state of health of the mucous membranes and how these alterations can play a role in the early diagnosis of serious diseases such as OSCC [58].

Most studies have focused their attention on the microbiome analysis, but the mycobiome is also connected to oral health and disease. Mycetes, especially *Candida*, could play a role in periodontal disease and oral cancer; in addition, bacterial pathogens like *P. gingivalis* and *A. actinomycetemcomitans* interface with Candida. In fact, this last improves the invasiveness of *P. gingivalis* and *A. actinomycetemcomitans* and suppresses the fungal growth [59].

## 4. Periodontal Disease and Its Impact on Salivaomics

Periodontitis is a chronic inflammatory disease characterized by irreversible periodontal attachment loss and alveolar bone destruction as a result of inflammation of the periodontium [60]. The World Health Organization reported that periodontitis is one of the main causes of tooth loss [61].

Like other chronic infectious-inflammatory-immune diseases, periodontitis is associated with considerably higher risks of adverse cardiovascular events [62]. Moreover, the daily life activities or professional interventions can lead to bacteremia because of the mioral cavity microorganisms [62].

There is a balance between the oral microbiome and individual immunity, when some factor alters this homeostasis, particularly in more sensitive subjects, it triggers the inflammatory process and consequently the destruction of the supporting tissues of teeth, so it represents a “collateral damage” [60].

Traditionally periodontitis is diagnosed using radiography and clinical measures of probing pocket depth (PD), bleeding on probing (BOP), and clinical attachment level (CAL). However, given that there are both molecules host-derived and bacteria-derived in saliva, which express inflammation, soft and hard tissues destruction, these last can be used as biomarkers for diagnosis, progression, and response to treatment of periodontal disease [63]. A biomarker is “any substance, structure, or process that can be measured in the body or its products and that can influence or predict the incidence of outcomes or diseases” [64].

The possible biomarkers (Table 1) for the periodontal disease are connected to the disease pathogenesis, so infection, inflammation and destruction of the supporting tissues thereby each biomarker is studied with his respective -omics approach [65].

Proteomic technology, an instrument for identifying protein/peptide biomarkers, is also applied for periodontitis [69,70]. There are different salivary group biomarkers which change in volume in periodontal patients’ saliva (Table 1): immunoglobulin (Ig), which neutralizes pathogenic bacteria [66], has a higher concentration in affected subjects [67]; inflammatory proteins like C-reactive protein (CRP), cytokines, IL-1, TNF-α, chemokines, growth factors, or bone metabolism-related cytokines (i.e., RANK/RANKL/OPG); metalloproteinases (MMPs) especially MMPs 8 and 9; nonspecific biomarkers like albumins, amylases, mucins, lactoferrins, lysozymes, histatins, or proteins related to oxidative stress (OS) are altered [68]. 

As it was mentioned above, there are host-derived biomarkers and bacteria-derived biomarkers [69]; these last include DNA and proteins, such as the activity of dipeptidyl peptidase IV, an enzyme connected to collagen degradation associated with periodontitis and *P. gingivalis* [63].

Metabolomics is a key to studying the pathophysiology of many diseases, in fact metabolites are small molecules resulting from endogenous catabolism or anabolism, so they are a mirror of the biological processes and they represent a good biomarker to investigate disease states [71]. Since saliva is simply sampled and expresses the pathologic molecular change of periodontal disease [72], it is possible to employ salivary metabolomics to detect small molecules involved in periodontitis such as products of the periodontium destruction and altered microbiome [73]. 

Kim et al. analyzed five metabolites that are end products of bacterial metabolism: ethanol, taurine, isovalerate, butyrate, and glucose and they studied their change in periodontal disease; the concentrations of taurine, isovalerate, butyrate, and glucose were considerably augmented in patients with periodontal disease, in contrast, ethanol concentration decreased very much consequently to bacterial metabolism [74]. 

miRNAs also are one of the “omics” constituents of salivaomics. There are many studies about their expression in periodontitis and their possible application like biomarkers of periodontal disease; however, the results are very heterogeneous [75]. A great advantage is that miRNAs are more stable than proteins or mRNAs in different types of samples [76]. The importance of this type of biomarker is due to their contribution in maintaining the cellular homeostasis [77], in fact, they are involved in numerous functions, like cell growth, proliferation and apoptosis [78].

Fujimori et al. have studied the correlation between salivary miRNAs and chronic periodontitis, and they show that salivary hsa-miR-381-3p may indicate periodontal disease [79]. Kang et al. demonstrated that the usage of salivary miR-23a and miR-146a as biomarkers for periodontitis still has some limits [80]. Five miRNAs are verified in multiple studies, as miR-142-3p, miR-146a, miR-203 and miR-223 [75].

The literature shows that miRNAs represent encouraging biomarkers thanks to their high diagnostic, prognostic and therapeutic potential, but they still need to be defined and investigated [27].

## 5. Salivaomics and Premalignant and OSCC

OSCC is one of the most widespread cancers in the world, in fact, in order of prevalence, it is the sixth most common cancer, so early detection and diagnosis becomes essential to improve the prognosis. Currently, oral cancer diagnosis relies on clinical examination and histological exam through biopsy. Since oral cancer is often difficult to detect because of the expression that is often asymptomatic and localized in hard-to-find regions, it becomes essential to comprehend the molecules involved in the pathogenesis of this disease to develop new strategies and therapies [45,81]. Moreover, the 5 years of survival of OSCC in stage 1 and stage 2 is about 85%, but in stage 3 and stage 4 still remains low, about 25% [82], so the early diagnosis is the crucial factor in improving the patient’s survival and to reduce the extension of the surgical wound [83]. 

In this regard, the opportunity to search for cancer biomarkers in body fluids is possible because the cancer-related mutations are detectable also distant from the tumor’s point of development [84], even more so, saliva represents a perfect biofluid because of its distance from the tumor. In fact, despite the salivary biomarkers concentration is less compared to bloodstream concentration, it is not a limitation because the oral cancer biomarkers are released directly in saliva [85,86].

It is possible to categorize the oral cancer biomarkers in the basis of the molecules like DNA, RNA or proteins and between diagnostic or prognostic biomarkers [87]. 

Many proteins have been investigated in saliva for the early diagnosis of oral cancer both separately and as a panel, since their detection may be useful for early diagnosis and control of tumor progression [88]. 

Among the most studied proteins in OSCC there are interleukin 8 (IL-8), IL-6, IL-1β, matrix metalloproteinase (MMP2, MMP9), transferrin, α-amylase, tumor necrosis factor alpha (TNF-α) and catalase [89]. 

Interleukins (IL-s) are a family of well-researched proteins because they control cellular migration, differentiation and apoptosis [69]. Particularly, IL-8 is a pro-inflammatory cytokine involved in tumor angiogenesis, cell adhesion, and cell cycle arrest [87]. Even though it is elevated in periodontal disease, IL-8 levels are much higher in the saliva of oral cancer patients [81].

The proangiogenic and pro-inflammatory cytokines IL-1, IL-6, IL-8 and TNF-α are elevated in saliva samples of patients with oral cancer and oral precancer, so they have the potential of surrogate indicators of carcinogenic mutation from oral precancer to oral cancer. Moreover, salivary IL-6 and TNF-α alterations may be implicated in the pathogenesis of oral Leukoplakia [90]. Goldoni et al. agree that IL-6, IL-8, IL-1α and IL-1β are all strong salivary biomarkers for oral cancer detection [7] (Table 2).

Although proteomic represents the first salivary diagnostic biomarker alphabet, also genomic targets are fundamental to investigate [90]. Genome is studied through high-throughput microarray technology, and in 2004 have been discovered more than 1600 altered genes involved in OSCC [91]. There are somatic mutations of tumor-specific DNA linked with carcinogenesis that represent specific biomarkers detectable in saliva [92]. In addition, it is possible to detect viruses DNA related to oral tumor like human herpes virus and HIV [93].

Dysregulation of miRNAs has a strong impact on cell growth, differentiation and apoptosis, in fact they may have a role as oncogenes or tumor suppressors [87] and genetic alterations, such as single nucleotide polymorphisms (SNPs), are connected to tumor prognosis and progression and to oral premalignant lesions (OPML) development. There are SNPs correlated to increased risk of oral cancer like rs3746444 in mir-499, rs2292832 in miR-149 and rs14035 in Ran, and there are SNPs connected to decreased risk of oral cancer such as rs11614913 in miR-196a2, rs2187473 in mir-34b and rs1057035 in DICER1 [94].

Park et al. show that the concentration of miR-125a and miR-200a is smaller in patients with OSCC [95]; in fact, miR-125a acts as a tumor suppressor in breast cancer cell lines the reduction of ERBB2 and ERBB3 levels [96]. Patients affected by OSCC have been shown to have increased salivary expression profiles of miR-31 and decreased miR-200a. In particular, monitoring miR-31 salivary levels may be useful for earlier OSCC detection since its concentrations are elevated at all clinical OSCC stages without alteration in oral potentially malignant lesions and healthy mucosa. the use of miRNAs as early biomarkers is still under study, but the preliminary results are very promising for interesting future developments [97].

OSCC is sometimes the evolution of some lesions named oral potentially malignant disorders (OPMDs) characterized by an increased risk for malignant transformation in a range from 0% to 20% in 1–30 years compared to the healthy oral mucosa. Leukoplakia, erythroplakia, oral lichen planus and oral submucous fibrosis are among the most common potentially malignant disorders [88].

### 5.1. Oral Lichen Planus

Oral lichen Planus (OLP) is a chronic immune-mediated inflammatory disease of oral soft tissues. It is a potentially malignant disease, representing a pathology of great interest with a 1.14% probability of cancer evolution. OLP is characterized by abnormal immune system activation that is reflected in circulating signal molecule concentration. Cortisol is a glucocorticoid involved in immunoregulation processes. Its concentrations are higher in saliva samples of patients with oral lichen planus; high cortisol levels are present in stress conditions that can trigger autoimmune disease. Also, oxidative stress-related molecules, such as nitric oxide (NO) and reactive oxygen species (ROS), are elevated in the saliva of patients with OLP [98]. Moreover, inflammatory cytokines like TNF-α, which mediate autoimmune and inflammatory processes, have been studied and recognized as potential biomarkers of this pathology [99]. IL-6, IL-8, IL-17, IL-18, IFN-γ, and TNF-α are all elevated in the saliva of patients with Oral Lichen Planus. Given the fact that there is a close linkage between oncogenesis and chronic inflammation, the altered concentration of pro-inflammatory cytokines in OLP could represent a pro-oncogenic factor [99,100] (Table 2). The expression of miR-155 in plasma of OLP patients was observed to be higher than in healthy controls, and the plasma concentrations of miR-155 and miR-146a were significantly higher in patients with erosive forms of OLP compared to subjects with non-erosive OLP, indicating the potential role of this molecules in the prediction of the severity of the disease [101].

### 5.2. Leukoplakia

Leukoplakia is a whitish lesion of oral mucosa that is impossible to remove by scraping, whereas histologically, it is possible to assess the risk of malignant transformation by looking at signs of cellular dysplasia like pleomorphism, myoepithelial basocellular hyperplasia, asymmetrical epithelial stratification, dyskeratosis and hyperchromatic nuclei [98].

Given the fact that it is one of the most widespread potentially malignant lesions of the oral cavity and that around 11% of squamous cell carcinomas derive from the malignant transformation of leukoplakia [102], it is interesting to find new technologies to detect this condition in addition to classic biopsy. 

Salivary TNFα has been studied as a possible biomarker for leukoplakia; it has been shown that TNFα concentrations are elevated in the saliva of patients with dysplasia [103]. Many authors have been focusing on the role of interleukins and on their concentration in saliva samples of a patient with leukoplakia and IL-6, and 1L-8 are highest in people affected than in healthy people [104] (Table 2).

Oncogenic miRNAs, miR-21, miR-181b and miRNA-345 are connected to the severity and malignant transformation of leukoplakia to oral cancer, in fact, they are overexpressed in this pathology [94].

**Table 2:** Change in concentration in saliva of biomarkers of OSCC, OLP and Leukoplakia.

Disease	Biomarker	Changes	
**OSCC**	IL-1	↑	[81]
**OSCC**	IL-6	↑	[89]
**OSCC**	IL-8	↑	[90]
**OSCC**	TNF-α	↑	[89]
**OLP**	IL-6	↑	[99,100]
**OLP**	IL-8	↑	[99,100]
**OLP**	TNF-α	↑	[99,100]
**OLP**	cortisol	↑	[98]
**OLP**	Nitric Oxide (NO)	↑	[98]
**OLP**	Reactive Oxygen Species (ROS)	↑	[98]
**Leukoplakia**	TNF-α	↑	[103]
**Leukoplakia**	IL-8	↑	[104]
**Leukoplakia**	IL-6	↑	[104]

## 6. Discussion

Periodontitis and oral cancer are two cornerstones of oral pathology, in fact the first is considered the major oral disease that leads to tooth mobility, tooth loss and masticatory dysfunction, so it represents a chapter of public health prevention [105]. On the other hand oral cancer, particularly OSCC, is a multifactorial disease in which male sex, old age, HPV, oral bacteria, ultraviolet radiations, betel-quid chewing are all risk factors of carcinogenesis with alcohol and tobacco which have a synergistic effect [7].

For this reason, it is important to have a rapid and effective sample for the diagnosis and management of these diseases. Salivary diagnostic is now a well-known diagnostic tool, and many efforts are spent on improving its diffusion and its clinical application.

Saliva is simple, rapid, and painless to obtain, and this makes it a perfect biological sample. Saliva, like other biofluids, has a specific composition, so it necessitates “fluid specific” devices to be collected and analyzed [106].

Every day 0.5 to 1.5 L of saliva are produced unless there are specific diseases and conditions [107]; in fact, different elements can alter saliva sample and its diagnostic potentials, such as patient’s age and gender and the method of collection [108,109].

There are two principal methods of saliva collection: unstimulated saliva and stimulated saliva, harvested in different ways. Stimulated saliva provides diluted samples, so it is less favourable than the unstimulated one. A guide called “Saliva Collection Handbook” explains how to properly collect saliva [110]. A correct collection’s procedure has to avoid the saliva contamination or the presence of substances that can alter the sample, like food, alcohol, caffeine and nicotine [7].

Saliva’s biomarkers can be detected by point-of-care (POC) devices or by other standard technologies [110]. It is possible to study DNA, mRNA or inflammatory cytokines through RT-PCR or Elisa. Moreover, proteins and peptides can be quantified with many biochemical techniques such as capillary electrophoresis, gel electrophoresis, magnetic resonance, liquid chromatography and immunoassay tests [111]. Commercial Elisa kits have good sensitivity, but they have a long data processing; now, the development of biosensors allows quicker and easier protocol: these are specific devices able to convert biological markers into measurable signal, as explained in Figure 3 [7]. Salivary “omic” technology has great clinical potential, but knowledge still needs to be expanded to apply it to clinical use.

## 7. Future Perspectives 

Saliva represents the present and future of clinical diagnostics. In fact, if one often-cited criticism of using saliva as a diagnostic fluid is that biomarkers are present in amounts that are too low to be detected reliably, on the other side, the introduction of new sensitive technologies able to analyze small quantities of molecules in body fluid and the deployment of standardized saliva collection devices, saliva is becoming an effective and predictable diagnostic tool [112]. The term “salivaomics” underlines all the technologies used to study salivary biomarkers such as transcriptomics, genomics, microRNA, proteomics, and metabolomics [110]. Saliva as a diagnostic biofluid has many clinical advantages and does not require specialized medical staff, and therefore, there is a reduction in healthcare costs [19]. Despite all these potential, salivary diagnostic still lacks effective clinical application because salivary biomarker undergoes variations with the circadian cycle and are influenced by diet and environment, and standardized salivary molecular identification systems need to be improved [113]. Salivary “omic” technology has great clinical potential, but knowledge still needs to be expanded to apply it to clinical use.

## 8. Conclusions

Saliva provides a good diagnostic tool, and undoubtedly saliva collection is simple and rapid also for the general dentist. Lots of novel technologies have indeed been developed, but salivary diagnostic still lacks real clinical application. It is due of the fact that specific biomarkers have not been found yet. In fact, periodontitis and oral cancer are multifactorial diseases and the biomarkers we talked about are general index of inflammation that cannot be considered diagnostic alone. 

Surely scientific research is interested in finding more biological markers in this field of medicine for the spread of periodontitis and oral disorders. However, the current knowledge on this topic is still limited, and further studies are needed to understand the role of salivaomics in dentistry better. 

## Figures and Tables

**Table 1:** Change in concentration in saliva of biomarkers of periodontal disease.

Disease	Biomarker	Changes	Mechanism	
**Periodontitis**	IL-1β	↑	support bone destruction	[66]
**Periodontitis**	IgA	↑	Inhibition of bacterial adhesion and activity	[66]
**Periodontitis**	MMP-8	↑	destruction of the supporting tissues	[67]
**Periodontitis**	Nitric Oxide (NO)	↓	Regulation of immune and inflammatory cell cellular mediating	[68]
